# AQUILA: assessment of quality in lower limb arthroplasty. An expert Delphi consensus for total knee and total hip arthroplasty

**DOI:** 10.1186/1471-2474-12-173

**Published:** 2011-07-22

**Authors:** Bart G Pijls, Olaf M Dekkers, Saskia Middeldorp, Edward R Valstar, Huub JL van der Heide, Henrica MJ Van der Linden-Van der Zwaag, Rob GHH Nelissen

**Affiliations:** 1 Department of Orthopaedics, Leiden University Medical Center, Leiden, The Netherlands, Albinusdreef 2, Room J-09-127; 2300 RC, Leiden, The Netherlands; P.O. Box 9600, Postzone J-11-S; 2300 RC Leiden, The Netherlands; 2 Department of Clinical Epidemiology, Leiden University Medical Center, Leiden, The Netherlands; 3 Department of Biomechanical Engineering, Faculty of Mechanical, Maritime and Materials Engineering, TU Delft, The Netherlands

**Keywords:** Total Knee Arthroplasty, Total Hip Arthroplasty, Reporting Quality, Methodological Quality, Generalizability

## Abstract

**Background:**

In the light of both the importance and large numbers of case series and cohort studies (observational studies) in orthopaedic literature, it is remarkable that there is currently no validated measurement tool to appraise their quality. A Delphi approach was used to develop a checklist for reporting quality, methodological quality and generalizability of case series and cohorts in total hip and total knee arthroplasty with a focus on aseptic loosening.

**Methods:**

A web-based Delphi was conducted consisting of two internal rounds and three external rounds in order to achieve expert consensus on items considered relevant for reporting quality, methodological quality and generalizability.

**Results:**

The internal rounds were used to construct a master list. The first external round was completed by 44 experts, 35 of them completed the second external round and 33 of them completed the third external round. Consensus was reached on an 8-item reporting quality checklist, a 6-item methodological checklist and a 22-item generalizability checklist.

**Conclusions:**

Checklist for reporting quality, methodological quality and generalizability for case series and cohorts in total hip and total knee arthroplasty were successfully created through this Delphi. These checklists should improve the accuracy, completeness and quality of case series and cohorts regarding total hip and total knee arthroplasty.

## Background

Observational studies (case series and cohorts) provide an important source of knowledge on total hip arthroplasty (THA) and total knee arthroplasty (TKA). In addition to personal experience, they are the most common type of evidence used by orthopaedic surgeons for clinical decision making according to a survey of the participants at the 2007 Annual Meeting of the American Orthopaedic Association [1].

Nevertheless, their rank in the hierarchy of scientific evidence is lower than evidence obtained from randomised experiments, and they often suffer from lack of a control group, incomplete data collection, selection bias and confounding by indication [2]. Despite these issues, case series and cohorts are important in signalling inferior prosthesis designs, particularly those prone to aseptic loosening, which accounts for 60% of THA revisions. They are therefore a valuable addition to clinical trials and implant registries [3-6]. Further advantages are great detail, relatively low costs, short study completion time and a potentially high external validity due to the inclusion of a wide range of patients [2].

Considering the substantial value and large volume of case series and cohorts in orthopaedic literature as well as the methodological issues mentioned above, it is remarkable that there is currently no validated measurement tool to appraise their quality [7]. A validated measurement tool could contribute to more accurate, transparent and complete case series and cohorts, resulting in higher quality [8]. Although STROBE is available as a guideline for reporting in observational studies it lacks details that are important for TKA and THA such as details on type of implant and surgical technique. Additionally, the STROBE-group has recently emphasized that STROBE is a reporting guideline and that it should not be misused for the appraisal of methodological quality [9].

The aim of this study was therefore to develop a tool to appraise the reporting quality and methodological quality of case series and cohorts of lower limb arthroplasty with emphasis on revision for aseptic prosthesis loosening by means of a Delphi approach. The second aim was to construct a checklist of items that are important for the generalizability of the results of case series and cohorts.

## Methods

A Delphi approach was used for the development of a checklist for reporting quality, a checklist for methodological quality and a generalizability tool. The Delphi approach is a well recognized research method for consensus formation amongst a group of experts through several iterations of questionnaires [10,11]. The advantages are anonymity of the participants, so avoiding dominance, expression of consensus by summary measures and several iterations with controlled feedback, which allows individuals to change their opinion in light of the group's response. A Delphi takes full advantage of both the research and clinical experience of the involved experts while imposing no geographical limitations on participation [10].

### Design of Delphi

An internet-based Delphi design was adapted from Graham et al. and the reporting was according to the CHERRIES guidelines for reporting results of internet E-surveys [12,13]. The focus of the Delphi was on the revision rate for aseptic loosening in TKA and THA. During the conceptual phase we determined that the checklists should require quality items (internal validity) and generalizability items (external validity) specific for TKA and TKA. Furthermore the quality items should include items for the appraisal of selection bias, confounding by indication and competing events [2,14]. Additionally, the checklists had to be easy to use, be able to be completed in an acceptable amount of time and had to allow for the possibility that items be scored as "unknown" in cases with insufficient information.

A master list of relevant items was created as a pre-checklist to allow external experts to asses the face validity and to further develop the final checklist through a Delphi method in an efficient fashion with the desire to optimize the construct validity. This kind of approach is common for consensus development through a Delphi [15-17]. The master list was generated from items of a recent systematic review of the literature and from the Equator Network website http://www.equator-network.org/ [18,19]. The authors of the manuscript, the internal working group, achieved consensus after evaluating and revising this master list in two internal rounds. The actions of the internal working group consisted of the rephrasing of selected items, so that these items met the requirements described above. Since item generation for the master list is an important initial step that may determine the course of the Delphi, we ensured that the members of the internal working group covered all fields (TKA, THA and epidemiology) of the Delphi, that no items were discarded during the internal rounds and that the master list was as comprehensive as possible. Additional aims of the internal rounds were completion of the master list and further testing and fine tuning of the web-based Delphi survey form. During the external rounds of the Delphi survey the internal working group analyzed and discussed the external experts' answers after each round, modified the list of items accordingly and rephrased, merged and clarified individual items to optimize their clarity and conciseness.

The Delphi survey consisted of three external rounds and the external experts consulted were not involved in the internal rounds and did not take part in the development of the survey [16]. In accordance with the principles of a Delphi survey each expert remained blind to the identity of other experts. The experts who completed the first external round were invited to participate in the second and third external rounds. During the second and third round the experts received a newly created checklist which was modified according to the results of the preceding round. Each item of the newly created checklist was presented with a summary of the groups' response to allow the experts change their answer in view of the groups' response [13].

Invited experts were identified via Pubmed and were required to have had at least one international peer-reviewed publication in the last three years in the field of TKA, THA or evidence based medicine in more general terms (expertise in musculoskeletal field or reporting guidelines or advised by one of the authors). One reminder was sent to those experts who did not respond during the first external round. Four reminders were sent to non responders during the second and third external rounds. The reminders consisted of a personal e-mail message sent by the internal experts when applicable, in order to maximize the response rate [20]. The first internal round commenced in July 2009 and the last external round was concluded in June 2011.

### Design and handling of the E-survey

An electronic form was created in Google documents comprising 50 items in the first internal round, 42 items in the second internal round, 45 items in the first external round, 48 items in the second external round and 22 items in the third external round (only generalizability). The survey consisted of general items (e.g. expert name; remarks boxes), quality items and generalizability items.

External experts were invited by e-mail to complete the online survey. This e-mail contained a link to the survey, information regarding the purpose of the Delphi and an estimate of the duration of the survey as derived from the internal rounds. Experts were informed that they would be invited for further rounds before opening the survey. The only incentive used was an offer to the external experts of a mention in the acknowledgements on the condition of completion of two rounds.

All items of the survey, except the remarks boxes, were required items. Omitted questions were highlighted in cases with an incomplete submission. The survey consisted of a mixture of multiple-choice and open questions and included text boxes for remarks in order to take full advantage of the knowledge of the expert panel and to ensure creativity of the items. Furthermore all the multiple-choice questions in the first external round had the "other" option with a free text field, so that no restrictions were placed on the answers of the experts. Additionally, opportunity was given to the experts to add items, to modify wording of items and to give explanations and reasons for their answers. Text boxes for remarks ensured that experts could make additions, suggestions and remarks in an unrestricted manner.

Each expert had to answer all questions. Since the survey comprised multiple areas of expertise the experts could choose the option "*no opinion*" when necessary. Experts were able to view and change their answers before submission.

Experts were also asked for their names and e-mail addresses in order to prevent duplicate entries from the same individual.

### Domains of the Delphi

The three domains of the Delphi checklist were reporting quality, methodological quality and generalizibility.

#### Reporting quality and methodological quality

The Delphi distinguished between reporting quality and methodological quality, because while reporting quality is particularly important for transparency, methodological quality is helpful in appraising and understanding the sources and magnitude of bias in a study [9]. Accordingly, a study with a high level of reporting quality may be methodologically unsound (low methodological quality) and vice versa.

#### Generalizability

The fact that two studies will never be completely identical poses difficulties for the comparability and generalizability of their results [21]. Since patient demographics, component positioning, post operative functioning (activity level) and regional influences may all affect revision rates for aseptic loosening, so it is important to investigate to what extent each factor may differ between two studies [5,22-24]. For example, are the results of a study with 60% female patients comparable to those of a study with 90% female patients when all other factors are the same? Does each factor need to be exactly the same or are small differences acceptable and if so, to what extend? In order to identify relevant items, the experts were asked to select items that are important for case series and cohorts with aseptic loosening in TKA and THA. When an item was chosen they were then asked to specify the extent of the allowable difference, for each relevant factor, that would be acceptable when comparing different studies in terms of generalizibility.

### Statistical analysis

Standard descriptive statistics were used. For an item to be included in the final checklists it must have been selected by at least two thirds of the experts [25].

For generalizibility items the mode was determined, which is the value that was chosen most frequently (e.g. 5 years). The preference for the mode value was calculated by dividing the number of experts who chose the mode value by the total number of experts who considered the generilizability item relevant (N_Mode/_N_Total _) The preference was considered high in case 80% or more of the experts chose the mode value. The preference was considered moderate in case 67% to 80% of the experts chose the mode value and the preference was considered low in case fewer than 67% of the experts chose the same value.

The "*no opinion*" answers were not used for the calculation of agreement, because this option could be used by experts when faced with a question outside the scope of their expertise.

## Results

### Delphi flow

**Figure 1:**
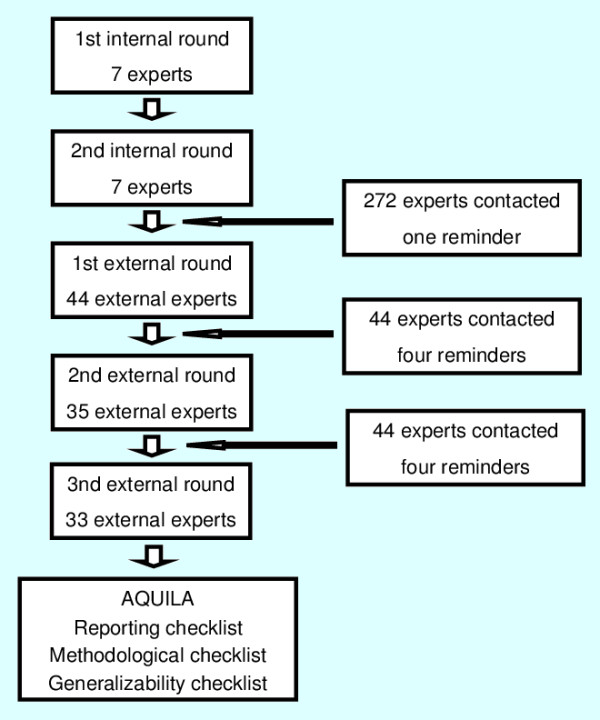
**Flowchart**. Overview of the Delphi flow and the number of experts involved in each round.

An overview of the Delphi flow and the number of experts involved in each round is depicted in Figure 1. Of the 272 experts contacted, 44 agreed to participate and completed the first external round. 37 of them also completed the second (n = 35) or third (n = 33) external round. These 37 external experts form the basis of this Delphi and had a mean experience of 16 years (range 3 to 30 years; S D7.5), see Table 1 for the area of expertise. The professional background of the experts was as follows: 30 orthopaedic surgeons or residents, 5 epidemiologists, 1 biomedical engineer and 1 physical therapist. The mean number of publications for all expert was 80 (range 2 to 445). The experts were of the following 17 nationalities covering 5 continents: American, Argentinean, Australian, Austrian, Belgian, British, Danish, Dutch, Finish, French, German, Indian, Israelian, Italian, Spanish, Swedish and New Zealander. Additional characteristics of the experts are presented in Table 1. The mean total completion time for all external rounds was 32 minutes SD 13 (range 17 to 65 minutes). There were no apparent differences in ratings and answers between the experts who completed both external rounds and those who only participated in the first external round.

**Table 1:** Characteristics of the experts (n = **37**) who completed the external rounds

	Count
Area of expertise^a^	

• Hip surgery	24

• Knee surgery	20

• Evidence Based Medicine	11

• Other^b^	7

Background*	

• Academic	27

• Public	9

• Private	6

• Other^c^	2

^a ^Multiple answers for each expert are possible. Therefore the total is more than 37.

^b ^One expert indicated "Implant Biology" in the other field. The remaining 6 answers in the other field were in addition to "Hip surgery", "Knee surgery" or "EBM"

^c ^One expert indicated "Private Research Center" in the other field. The remaining answer in the other field was in addition to "Private Hospital".

### Reporting quality and methodological quality

At the beginning of round 1 the Delphi consisted of two domains as determined by the internal working group: quality (internal validity) and generalizability (external validity) After round 1 a clear distinction between reporting items and methodological items was made, as suggested by one of the external experts. The quality items were therefore allocated to either the reporting quality checklist or methodological quality checklist. Furthermore, the FU-quotient has been added to methodological quality item nr 3, as suggested by one of the external experts [26]. Additional modifications after round 1 consisted mainly of rephrasing. Some items were divided into two separate items (5 years post-operatively and 10 years post-operatively). Following round 1 these items were compiled into one item without a time specification. The responses of the experts to reporting items and methodological items in all rounds are presented in Additional File 1.

By the second external round, agreement was reached on eight items relating to reporting quality as well as on six items on methodological quality. Additionally, 21 of the 35 experts indicated that a case series or cohort should include at least 100 arthroplasties at baseline in order to accurately determine the number of revisions or revision rate. The answers ranged from a minimum of 40 to a minimum of 300 arthroplasties. The final list of items covering reporting quality and methodological quality can be found in Table 2.

**Table 2:** The final AQUILA checklist for use by authors

Reportinbg Quality Item
1. Are the inclusion and exclusion criteria clearly reported?

2. Is information regarding the number of patients who did not gave informed consent and who were not willing to participate adequately reported?

3. Are the baseline characteristics of included patients reported?

4. Is the surgical technique adequately reported?

5. Are the prosthesis brand and fixation reported with enough detail?

6. Are the reasons or definitions for revision adequately reported?

7. Are the number of revisions (N) and revision rates regarding aseptic loosening (either Kaplan-Meier or life table or revisions per 100 observed component years) adequately reported?

8. Is the number of deaths, lost-to-follow up (e.g. no show at clinic or emigration), amputations, and revisions other than the primary endpoint adequately reported?

**Methodological Quality Item**

1. Is there a clear primary research question/hypothesis?*

2. How were the cohorts constructed?
a. Consecutively^a^
b. Non-consecutively
c. Unknown

3. How adequate was the follow-up (FU)?
a. Fully completed FU
b. 5% or less lost-to-FU or FU quotient^b ^is 1 or less
c. More than 5% lost-to-FU or FU quotient is more than 1
d. Unknown

4. How was the FU performed?
a. Predefined e.g. yearly
b. When patients had complaints or chart review (of non-predefined FU)
c. Unknown

5. How many arthroplasties are at risk at the FU of interest?
a. 20 or more
b. Less than 20
b. Unknown

6. Has a worst case analysis or competing risk analysis for competing endpoints [14] been performed?

* In cases of aseptic loosening: Does the research question or hypothesis include revision of the component due to aseptic loosening?

^a ^Consecutively is defined as all patients receiving an arthroplasty (TKA or THA) in a defined period of time have also received the arthroplasty of interest. The following situation is therefore non-consecutive: patients receiving prosthesis X while prosthesis Y has also been used for the same indication during the specified period.

^b ^FU quotient = Number lost to follow up/Number of failures [26].

### Generalizibility

After round 1 the following items were dropped from the checklist, because less than two thirds of the external experts found them relevant: Hospital for Special Surgery Score (TKA), Merle D'Aubigné Score (THA) and Range of Motion (THA). After the second round the following items were added to the checklists, as suggested by one of the experts: KOOS (TKA), WOMAC (TKA), Oxford Knee Score (TKA), HOOS (THA), WOMAC (THA) and Oxford Hip Score (THA). All these six items were considered relevant in the third round and thus remained in the final checklist.

Twenty-two items, related to the comparison of revision rates between studies, were agreed upon by the third external round. These items comprised domains of patient demographics, component positioning, post-operative functioning and regional influences. The final list of these generalizability items can be found in Table 3 and details about the procedure are available in Additional File 2.

**Table 3:** Final list of generalizability items

Generalizability item		Mode^a^	N_Mode _of N_Total _(%)^b^	Preference for mode value^c^
Patient demographics				

Age		5 years	22 of 31 (71)	M

Gender		10%	20 of 30 (67)	M

Diagnosis		10%	17 of 31(55)	L

BMI		5 points	16 of 29 (55)	L

Component positioning				

TKA	Hip Knee Angle	5 degrees	13 of 24 (54)	L
	
	Varus/valgus tibial component	3 degrees	17 of 25 (68)	M
	
	Slope of tibial component	3 degrees	15 of 24 (63)	L

THA	Inclination of acetabular cup	10 degrees	19 of 28 (68)	M
	
	Varus/valgus femoral stem	5 degrees	16 of 27 (60)	L

Post-operative functioning				

TKA	Knee Society Score	10 points	18 of 23 (78)	M
	
	Knee Society Function Score	10 points	20 of 24 (83)	H
	
	Range of Motion	10 degrees	18 of 24 (75)	M
	
	KOOS	10 points	11 of 17 (65)	L
	
	WOMAC Knee	10 points	11 of 19 (58)	L
	
	Oxford Knee Score	5 points	18 of 24 (82)	H

THA	Harris Hip Score	10 points	17 of 21 (81)	H
	
	HOOS	10 points	12 of 17 (71)	M
	
	WOMAC Hip	10 points	12 of 20 (60)	M
	
	Oxford Hip Score	5 points	16 of 22 (73)	M

Regional influences				

Are the studies from the same region (developing country or western countries//continents)?				

Are the studies similar in type en experience of the surgeon (academic; high volume; consultant; trainee)?				

Are two studies similar regarding hospital type (developer hospital/special institute/regular hospital)?				

^A ^Mode: the value that was chosen most frequently (e.g. 5 years)

^b ^N_Mode _= the number of experts who chose the mode value

N_Total _= the total number of experts who considered the generalizability item relevant

^c ^H = High preference, 80% or more of the experts chose the mode value

M = Moderate preference, between 67% and 80% of experts chose the mode value

L = Low preference, less than 67% of experts chose the mode value

Example: the preference for the mode value "5 years" is moderate.

## Discussion

The AQUILA initiative resulted in a checklist for reporting quality, methodological quality and generalizability for case series and cohorts of total hip and total knee arthroplasty. The STROBE guidelines are already available for use in reporting original patient research in TKA and THA. The AQUILA checklist now adds to these guidelines, as a treatment specific extension of STROBE, addressing items that are specific for TKA and THA in observational studies. Additionally, the AQUILA checklist addresses both methodological quality and generalizability, while STROBE is strictly a reporting guideline [9]. Since there are currently no specific checklists available for the assessment of case series or descriptive cohorts in lower limb arthroplasty, nor in orthopaedics in general, the AQUILA checklists should have an important role in improving the accuracy, completeness and quality of TKA-and THA-related case series and cohorts [8].

In terms of generalizability, there was consensus on the items that are relevant when comparing revision rates between studies, although in round 3 most of the included postoperative functioning items only just reached the cut off point of two thirds, see Additional File 2. However, the preference for the mode values (e.g. 5 years) was mostly moderate and even low for some items. This was most notable for component positioning and some functional outcome scores and may be a reflection of the ongoing research into the development of a core set of outcome measures and the current controversy in literature regarding neutral alignment of prostheses [27,28].

We should also note some limitations. As mentioned above, although consensus was achieved on the relevance of the generalizability items, the preference for the mode value (e.g. 5 years) was mostly moderate and even low for some items. The latter should therefore be interpreted with some caution. Furthermore, the application of a pre-checklist may have dampened the creativity of the external experts. However, this approach has been successfully used in the development of other checklists [15-17].

The possibility that the results were affected by non-responder bias should also be considered. As is the case for all surveys, the responders may have different opinions to those of the non-responders. However, experts who participate in a survey can be very similar to those who decline, as demonstrated by a study from McKee et al [29]. Indeed, the final expert panel in our study consisted of a balanced sample representative of the international musculoskeletal scientific community involving 17 nationalities on five different continents and included experts with a wide range of experience (mean 16 years range 3 to 30 years). Furthermore, the face validity of the checklists was good and at least 88% of the experts with an opinion consider the reporting quality and methodological quality items relevant. Moreover, the experts were unanimous in 8 out of 14 items.

The participation rate was 44/272 (16%). This is towards the lower end of participation rates commonly achieved in this type of survey [20,30]. The number of experts who completed at least two external rounds (n = 37) is respectable, considering that some Delphi's are based on as few as 12 experts [11,13]. Our aim was to obtain a balanced and representative sample of experts thus minimizing bias due to the selection of a small group of experts with a particular opinion. This highly sensitive approach could therefore have resulted in a dilution of available and interested experts. Accordingly, the response rate of the first external round is the trade off for the representative and balanced sample of experts obtained in our study. Furthermore, as only complete responses were recorded, incomplete responses could have been missed. Nevertheless 44 experts responded to the first external round and the response rate in the second (80%) and third (75%) external rounds was high.

It is not uncommon that studies of the same type of TKA or THA report rather different revision rates [31]. What factors have caused this difference? Are dissimilarities in patient demographics the cause, or component positioning, or post-operative functioning or perhaps regional influences (including skill and experience of the surgeon)? The generalizabity checklist provides a tool to help address this issue. For example: if the difference in mean age between two study populations is lager than 5 years, age is considered an important factor according to the results of the AQUILA.

Although the name *Assessment of Quality in Lower limb Arthroplasty *may suggest otherwise, the AQUILA was developed specifically for THA and TKA, and does not include Total Ankle Arthroplasty (TAA) or other types of lower limb arthroplasty. However, some of the reporting and methodological quality items may also be useful for the appraisal of these types of lower limb arthroplasty studies, since the mechanisms of bias (e.g. selection bias and competing risks) are the same [2,14]. On the other hand, the recommended minimal number of arthroplasties at baseline (100) may not be realistic for TAA Studies. Some of the generalizibility items, especially regarding component positioning and post-operative functioning may also not be applicable to TAA studies.

While the AQUILA checklist was specifically developed for revision rates for aseptic loosening, it may also be useful for other endpoints in lower limb arthroplasty, such as revision rates for septic loosening or revision for other reasons, since the mechanisms of bias are the same [2,14].

## Conclusions

In conclusion, the AQUILA checklist is the first tool that can be used to assess the quality of reporting, methodology and generalizibility in case series and cohorts in lower limb arthroplasty. Use of the checklist will lead to more accurate, transparent and complete case series and cohorts in this field [8].

## Competing interests

The authors declare that they have no competing interests.

## Authors' contributions

The following authors designed the study BP, EV, HH, RN, SM, analyzed the data BP, HH, wrote the manuscript BP, HH, OD and ensured accuracy of data and analysis HL, RN, OD, SM. All authors were involved as internal experts for the creation of the master list, completed both two internal rounds and assisted during the external rounds. Critical revision of the manuscript was performed by all authors. All authors read and approved the final manuscript.

## Pre-publication history

The pre-publication history for this paper can be accessed here:

http://www.biomedcentral.com/1471-2474/12/173/prepub

## Supplementary Material

Additional file 1 **Experts' responses to the AQUILA reporting quality and methodological quality items**. Click here for file

Additional file 2 **Experts' responses to relevance of generalizability items**. Click here for file
