# Supplementary material for: AQUILA: assessment of quality in lower limb arthroplasty. An expert Delphi consensus for total knee and total hip arthroplasty
Source: BMC Musculoskelet Disord. 2011 Jul 22;12:173. doi: 10.1186/1471-2474-12-173 (PMC3155910; doi:10.1186/1471-2474-12-173)
Supplement: Additional file 1 — Experts' responses to the AQUILA reporting quality and methodological quality items. [file 1471-2474-12-173-S1.DOC]

Additional file 1: Expert’s responses to the AQUILA reporting quality and methodological quality items

| **Reporting Quality Item:** | I | Round 1 | | | Round 2 | | |
| --- | --- | --- | --- | --- | --- | --- | --- |
|  |  | R | NR | O | R | NR | O |
| 1. Are the in- and exclusion criteria clearly reported? | Y | 44 | 0 | 0 | 35 | 0 | 0 |
| 1. Is information adequately reported regarding the number of patients who did not gave informed consent and who were not willing to participate? | Y | 39 | 5 | 0 | 30 | 4 | 1 |
| 1. Are the baseline characteristics of included patients reported? | Y | 41 | 1 | 2 | 35 | 0 | 0 |
| 1. Is the surgical technique adequately reported? | Y | 40 | 2 | 2 | 35 | 0 | 0 |
| 1. Are the prosthesis brand and fixation reported with enough detail? | Y | 41 | 2 | 1 | 35 | 0 | 0 |
| 1. Are the reasons or definitions for revision adequately reported? | Y | 43 | 0 | 1 | 35 | 0 | 0 |
| 1. Are the number of revisions (N) and revision rates regarding aseptic loosening (either Kaplan-Meier or life table or revisions per 100 observed component years) adequately reported? | Y | 42 | 4 | 5 | 33 | 1 | 1 |
| 1. Are the number of deaths, lost-to-follow up (e.g. no show at clinic or emigration), amputations, and revisions other than the primary endpoint adequately reported? | Y | 41 | 2 | 1 | 35 | 0 | 0 |

I = included in final list: Y = yes; N = No; R = Relevant; NR = Not Relevant; O = No opin

| **Methodological Quality Item:** | I | Round 1 | | | Round 2 | | |
| --- | --- | --- | --- | --- | --- | --- | --- |
|  |  | R | NR | O | R | NR | O |
| 1. Does the research question or hypothesis include revision of the component due to aseptic loosening? | Y | 41 | 0 | 3 | 33 | 0 | 2 |
| 1. How were the cohorts constructed? | Y | 43 | 0 | 1 | 34 | 1 | 0 |
| 1. How was the adequacy of follow-up (FU)? | Y | 41 | 1 | 1 | 34 | 1 | 0 |
| 1. How as the FU performed? | Y | 41 | 1 | 1 | 34 | 1 | 0 |
| 1. How many arthroplasties are at risk at the FU of interest? | Y | 34 | 3 | 7 | 32 | 0 | 3 |
| 1. Is a worst case analysis or competing risk analysis for competing endpoints[24] performed? | Y | 27 | 8 | 9 | 26 | 2 | 7 |

I = included in final list: Y = yes; N = No; R = Relevant; NR = Not Relevant; O = No opinion
