# Supplementary material for: AQUILA: assessment of quality in lower limb arthroplasty. An expert Delphi consensus for total knee and total hip arthroplasty
Source: BMC Musculoskelet Disord. 2011 Jul 22;12:173. doi: 10.1186/1471-2474-12-173 (PMC3155910; doi:10.1186/1471-2474-12-173)
Supplement: Additional file 2 — Experts' responses to relevance of generalizability items. [file 1471-2474-12-173-S2.DOC]

Additional file 2: Expert responses to relevance of generalizability items

| Generalizability item | | I | Round 1 | | | Round 2 | | | Round 3 | | |
| --- | --- | --- | --- | --- | --- | --- | --- | --- | --- | --- | --- |
|  |  |  | R | NR | O | R | NR | O | R | NR | O |
| Patient demographics | | | | | | | | | | | |
| Age | | Y | 35 | 1 | 8 | 32 | 3 | 0 | 31 | 0 | 2 |
| Gender | | Y | 27 | 5 | 12 | 32 | 3 | 0 | 30 | 1 | 2 |
| Diagnosis | | Y | 34 | 2 | 8 | 31 | 3 | 1 | 31 | 0 | 2 |
| BMI | | Y | 23 | 7 | 14 | 29 | 4 | 2 | 29 | 1 | 3 |
| Component positioning | | | | | | | | | | | |
| TKA | Hip Knee Angle | Y | 24 | 4 | 16 | 25 | 3 | 7 | 24 | 2 | 7 |
|  | Varus/valgus tibial component | Y | 26 | 4 | 14 | 26 | 2 | 7 | 25 | 1 | 7 |
|  | Slope of the tibial component | Y | 28 | 3 | 13 | 25 | 3 | 7 | 24 | 2 | 7 |
| THA | Inclination of the acetabular cup | Y | 27 | 1 | 16 | 28 | 2 | 5 | 28 | 0 | 5 |
|  | Varus/valgus femoral stem | Y | 19 | 7 | 18 | 24 | 5 | 6 | 27 | 1 | 5 |
| Post-operative functioning | | | | | | | | | | | |
| TKA | Knee Society Score | Y | 20 | 11 | 13 | 20 | 9 | 6 | 23 | 4 | 6 |
|  | Knee Society Function Score | Y | 20 | 10 | 14 | 20 | 9 | 6 | 24 | 3 | 6 |
|  | Hospital for Special Surgery Score | N | 18 | 10 | 16 | 19 | 9 | 7 |  |  |  |
|  | Range of Motion | Y | 22 | 8 | 14 | 20 | 9 | 6 | 24 | 4 | 5 |
|  | KOOS | Y |  |  |  |  |  |  | 17 | 4 | 12 |
|  | WOMAC Knee | Y |  |  |  |  |  |  | 19 | 4 | 10 |
|  | Oxford Knee Score | Y |  |  |  |  |  |  | 22 | 3 | 8 |
| THA | Harris Hip Score | Y | 16 | 12 | 16 | 21 | 10 | 4 | 22 | 6 | 5 |
|  | Merle DÁubine Score | N | 13 | 17 | 14 | 15 | 14 | 6 |  |  |  |
|  | Range of Motion | N | 16 | 16 | 12 | 20 | 11 | 4 |  |  |  |
|  | HOOS | Y |  |  |  |  |  |  | 17 | 5 | 11 |
|  | WOMAC Hip | Y |  |  |  |  |  |  | 20 | 4 | 9 |
|  | Oxford Hip Score | Y |  |  |  |  |  |  | 22 | 3 | 8 |
| Regional influences^a^ | | | | | | | | | | | |
| Are the studies from the same region  (developing country or western countries // continents)? | | Y | 31 | 8 | 4 | 27 | 6 | 2 |  |  |  |
| Are the studies similar in type and experience of the surgeon  (academic; high volume; consultant; trainee)? | | Y | 35 | 4 | 4 | 31 | 2 | 2 |  |  |  |
| Are two studies similar regarding hospital type  (developer hospital/ special institute/ regular hospital)? | | Y | 36 | 4 | 3 | 31 | 1 | 3 |  |  |  |

I = included in final list: Y = yes; N = No;

R = Relevant; NR = Not Relevant; O = No opinion

^a^ Consensus for these items was reached in the second external round, so no third round was required.
